# Identification of Ovine Serum miRNAs Following Bacterial Lipopolysaccharide Challenge

**DOI:** 10.3390/ijms21217920

**Published:** 2020-10-25

**Authors:** Ankita Sharma, Umesh K. Shandilya, Tianna Sullivan, Danielle Naylor, Angela Canovas, Bonnie A. Mallard, Niel A. Karrow

**Affiliations:** 1 Department of Animal Biosciences, University of Guelph, Guelph, ON N1G 2W1, Canada; ankitas@uoguelph.ca (A.S.); ushand@uoguelph.ca (U.K.S.); tsulli03@uoguelph.ca (T.S.); dnaylor@uoguelph.ca (D.N.); acanovas@uoguelph.ca (A.C.); 2 Department of Pathobiology, Ontario Veterinary College, University of Guelph, Guelph, ON N1G 2W1, Canada; bmallard@ovc.uoguelph.ca

**Keywords:** lipopolysaccharide, microRNA, serum, lambs, expression

## Abstract

Host–pathogen interactions are complex and influenced by host genetic and epigenetic modifications. Recently, the significance of microRNAs (miRNAs) in pathogenic infection and the regulation of immune response has been highlighted. However, information on miRNAs’ role in the course of inflammation is still very limited in small ruminants. The present study was intended to identify changes in the expression of circulatory miRNAs post-lipopolysaccharide (LPS)-challenge. In this study, young ewes (*n* = 18) were challenged with *Escherichia coli* LPS (400 ng/kg *i.v.*) and blood samples were collected for serum miRNA isolation at two-time points; prior to challenge (T0), and 4 h (T4) post-challenge, reflecting the peak cortisol response. A total of 91 miRNAs were profiled, including 84 miRNAs on a commercial ovine miRNA-PCR array, and seven individual miRNAs. Forty five miRNAs were differentially expressed (DE) with 35 being up-regulated (Fold regulation, FR > 2) and 10 being down-regulated (FR < 1, *p* < 0.05) at T4. Among the up-regulated miRNAs, 14 were significantly (*p* < 0.05) induced, including oar-miRs: 369-3p, 495-3p, 376a-3p, 543-3p, 668-3p, 329a-3p, 655-3p, 411a-5p, and 154a-3p, which were located on ovine chromosome 18 forming four miRNA clusters within 10 kb. The elevated miRNAs belonged to different functional classes, playing roles in activating the hypothalamic-pituitary-adrenal axis; increasing cell survival and differentiation; and inducing inflammatory responses and targeted PI3K-Akt and MAPK signaling and chemokine signaling pathways. In summary, these results reveal the dynamic nature of ovine serum miRNAs during LPS-induced stress and highlight the potential role of identified miRNA-clusters on chromosome 18 to understand the regulation of the acute-phase response. Some of these identified circulating miRNAs may also serve as stress biomarkers for livestock in the future.

## 1. Introduction

Cross-talk between both neuroendocrine and immune systems occurs during microbial infection to regulate the effector response and help restore physiological homeostasis. Activation of the innate immune system during the acute-phase response, for example, triggers neuroendocrine responses such as fever and sickness. Activation of the hypothalamic–pituitary–adrenal (HPA) “stress” axis during immune system activation leads to a temporal increase in blood glucocorticoid (cortisol) concentration that helps to minimize potential collateral tissue damage caused by the host’s inflammatory response.

The innate immune system is activated by recognition of pathogen-associated molecular patterns (PAMPs) by host pattern recognition receptors (PRRs). Lipopolysaccharide (LPS), which is a potent PAMP making up the cell membranes of Gram-negative bacteria such as *Escherichia coli*, is primarily recognized by the PRR toll-like receptor 4 (TLR4). This PAMP contributes to several livestock pathologies, including mastitis [1], acidosis [2], and gut leakage due to heat stress [3]; and human pathologies, such as systematic inflammatory response syndrome and sepsis [4]. Our previous studies have utilized *E. coli* LPS as an acute stressor of sheep to characterize the stress response and determine the genetic contribution to variation in the stress response [5,6,7]. Several other reports have provided evidence of varying immune responses amongst sheep breeds, indicating varied genetic regulation of the innate and adaptive immune systems [8,9,10].

Complex host–pathogen interactions occur during disease initiation, development, and progression. The outcome of these interactions is influenced by genetic predisposition and environmental factors that affect gene expression, possibly via epigenetic mechanisms such as microRNA (miRNA)–protein complexes [11]. Recent studies have provided some insights into the critical participation of miRNAs during inflammation and for regulating immune cell development [12]. MicroRNAs are short non-coding RNAs (20–22 nts) that mainly arbitrate the post-transcriptional modification of gene expression and act as regulators of multiple functions, including reproduction, metabolism, stress, and immunity. Recently, studies have demonstrated the involvement of miRNAs in a range of conditions, such as liver and cardiovascular diseases, cancer, autoimmune diseases, and altered physiological states; and circulating miRNAs that are released as signaling molecules for cell to cell communication have been proposed as potential biomarkers for predicting risk for disease [13,14]. These miRNAs circulate via packaging into microparticles such as exosomes, microvesicles, and apoptotic bodies, and can also be transported by either RNA-binding proteins or high-density lipoproteins, all of which contribute to their remarkable stability in biofluids. Accumulating evidence suggests that differential expressions of circulatory miRNAs in vivo can reflect the status of physiological changes and serve as biomarkers for diverse physiological and pathological conditions [15,16]. The potency of LPS for inducing a systemic inflammatory response has been documented via assessment of aberrant changes in miRNA expression [6,17], thereby suggesting that LPS is suitable for examining the involvement of miRNA in regulating the acute phase response. Therefore, the present study aimed to investigate the expression patterns of ovine circulating miRNAs at the peak cortisol response during acute systemic LPS challenge; these miRNAs could be used as stress biomarkers for livestock and may help to better understand coordinated cross-talk between the neuroendocrine and immune systems.

## 2. Results

### 2.1. Ovine miRNAs Associated with LPS Stress Challenge

**Figure 1:**
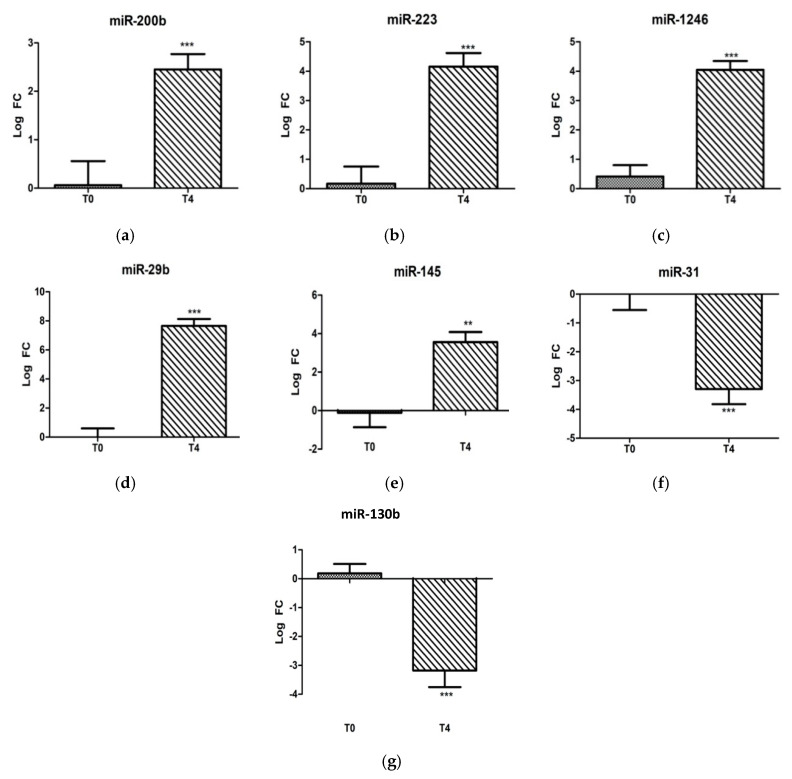
Fold-change expressions of 7 individually analyzed ovine microRNAs: (**a**) miR-200b, (**b**) miR-223, (**c**) miR-1246, (**d**) miR-29b, (**e**) miR-145, (**f**) miR-31, and (**g**) miR-130b at 0 and 4 h post-LPS-challenge (400 ng/kg i.v.). Significance between time points is denoted by (**) for *p* < 0.01 and (***) for *p* < 0.005.

To identify the miRNAs associated with the LPS stress challenge, the T4 post-challenge serum samples were compared with T0 pre-challenge samples. With regard to the individual candidate miRNA analysis, miR-29b, miR-1246, miR-223, miR-200b, and miR-145 were significantly induced (*p* < 0.05) post-challenge, and miR-31 and miR-130b were decreased significantly (*p* < 0.05) (Figure 1).

**Figure 2:**
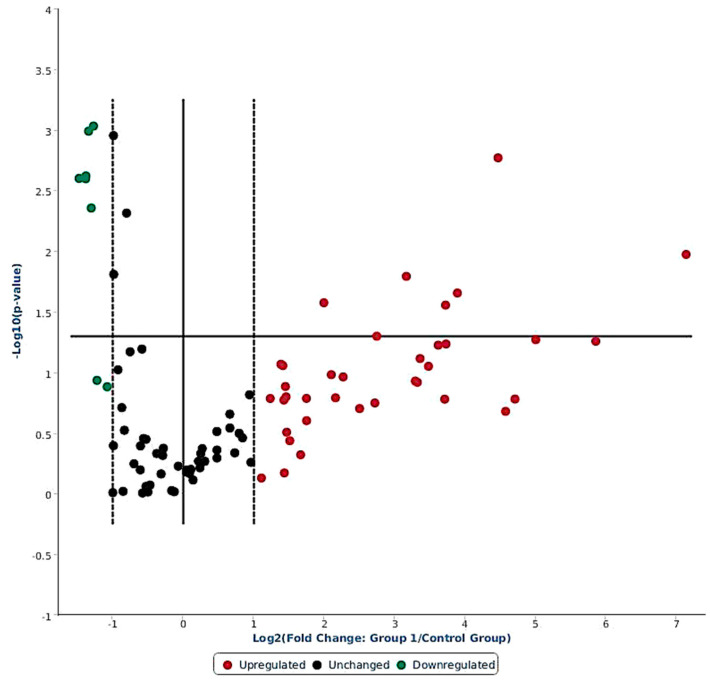
Volcano plot showing differential expression of 84 ovine miRNAs in response to systemic LPS challenge (4 h versus basal). The miRNAs having FR > 2 are on right hand side of the dotted line (red color), and miRNAs with FR < 1 are on left hand side of the dotted line (green color); whereas the miRNAs presented in between the two dotted lines had no change in expression (black color).

Out of 84 miRNAs expressed in the ovine miScript-PCR platform, there were 38 differentially expressed (DE) miRNAs having FR values > 2 (up-regulated) and FR < 1 (down-regulated) in response to LPS challenge; 40 miRNA remained unchanged, and 6 miRNAs were not expressed in any of the samples (Figure 2, Supplementary Table S1). Among 38 DE miRNAs, 30 were up-regulated (FR > 2) and 8 miRNAs were down-regulated (FR < S1). Out of the 30 up-regulated miRNAs, 9 miRNAs were significantly induced: oar-miR-369-3p (+140.9 fold, *p* < 0.05), oar-miR-495-3p (+57.9 fold, *p* < 0.05), oar-miR-376a-3p (+32.0 fold, *p* < 0.05), oar-miR-543-3p (+22.0 fold, *p* < 0.01), oar-miR-668-3p (+14.8 fold, *p* < 0.05), oar-miR-329a-3p (+13.0 fold, *p* < 0.05), oar-miR-655-3p (+9.0 fold, *p* < 0.02), oar-miR-411a-5p (+6.7 fold, *p* < 0.05), and oar-miR-154a-3p (+4.0 fold, *p* < 0.03). Among the down-regulated miRNAs, six were significantly (*p* < 0.05) reduced with FR < 1: oar-miR-380-5p, oar-miR-1197-5p, oar-miR-323b, oar-miR-665-5p, oar-miR-323c, and oar-miR-154b-3p (Table 1).

### 2.2. Genomic Localization and Cluster Analysis of Differentially Expressed (DE) miRNA

To further characterize the DE miRNAs in response to LPS stress challenge, the genomic distances between miRNAs were determined with respect to their locations in the *Ovis aries* genome (Oar_v4.0). The miRNAs were queried in miRbase for their chromosomal sequence sites. The encoded sequences of miRNAs were scattered across a total of five chromosomes: 18, 12, 14, 6, and X. Intriguingly, the all differentially expressed miRNAs (oar-mir-411a, oar-mir-329a, oar-mir-1197, oar-mir-154, oar-mir-485, oar-mir-376a, oar-mir-543, oar-mir-495, oar-mir-369, oar-mir-668, oar-mir-655, oar-mir-323, oar-mir-380, oar-mir-665) from ovine miScript-PCR platform were located on chromosome 18. On the other hand, the individually analyzed candidate miRNA were found on different chromosomes: mir-200b and mir-29b on chromosome 12, mir-1246 and mir-223 on the X chromosome, mir-31 on chromosome 14, and mir-130b on chromosome 6.

**Figure 3:**
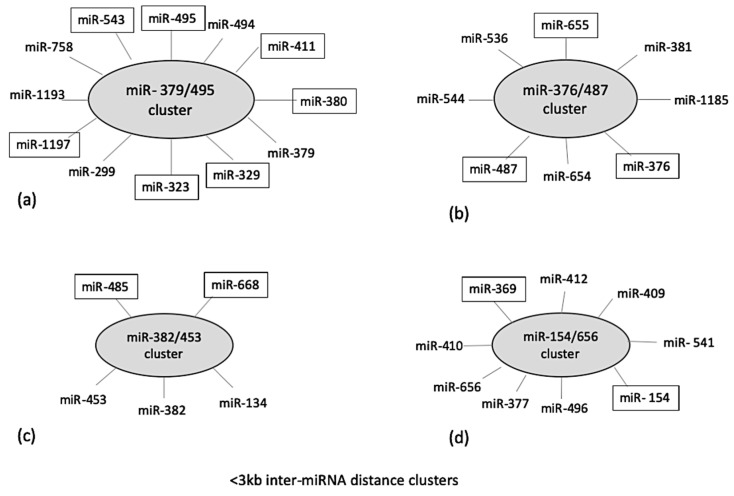
Cluster analysis of identified differentially expressed miRNAs at 4 h post-LPS-challenge, using two different databases: miRbase and MetaMirClust at <3000 bp inter-miRNA distance. The miRNAs highlighted (in boxes) in given clusters were differentially expressed in the present study. The total length of cluster (**a**) is 11,450 bp; cluster (**b**) is 12,003 bp; cluster (**c**) is 1888 bp; and cluster (**d**) is 6729 bp.

Localization of identified miRNAs on a common chromosome can result in a cluster formation which has been reported to co-regulate different biological processes [18]. Therefore, cluster analysis was performed for the DE ovine miRNAs using miRbase and MetaMirClust. Among these miRNAs, four different miRNA clusters were identified based on an inter-miRNA distance of less than <3000 bp on the same genomic strand. These identified clusters were miR-379/495 consisting of five DE miRNAs, miR-376/487 consisting of three DE miRNAs, miR-382/453 consisting of two DE miRNAs, and miR-154/656 consisting of two DE miRNAs (Figure 3). The MetaMirClust program further stated that clusters miR-411/329 and miR-376/655 were conserved across various species (humans, cows, horses, guinea pigs, dogs, and mice) (data not shown).

### 2.3. Gene Enrichment and Pathways Analysis

**Figure 4:**
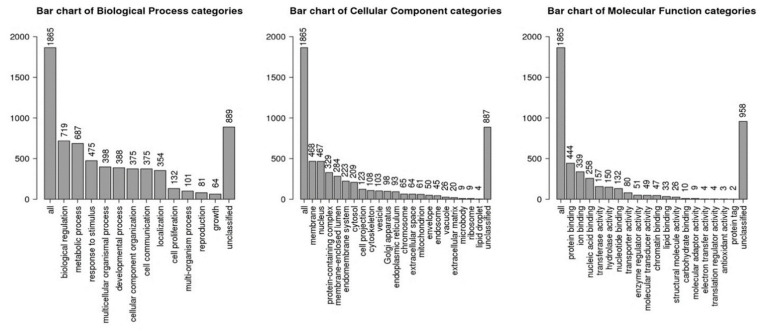
Gene ontology (GO) terms identified by identified target genes of upregulated miRNAs using Web-GSTALT online database in three categories: biological processes, molecular functions and cellular components.

A total of 1966 target genes were identified for 15 up-regulated miRNAs using three different prediction tools (miRDb, Miranda, TargetScan). Functional gene enrichment analysis of the predicted target genes using WeB-Gestalt webserver is shown in Table 2. The top significantly enriched terms in the molecular function category were: regulation of transcription activity (GO:0140110, False discovery rate, FDR- 1.57 × 10^−8^), DNA binding transcription factor activity (GO:0003700, FDR- 1.64 × 10^−8^), RNA polymerase II regulatory region sequence-specific DNA binding (GO:0000977, FDR- 2.41 × 10^−8^), regulatory region nucleic acid binding (GO:0001067, FDR- 3.16 × 10^−8^), and transcription regulatory region sequence-specific DNA binding (GO:0000976, FDR- 3.16 × 10^−8^). The top significantly enriched terms in the biological processes category were: regulation of gene expression (GO:0010468, FDR- 1.48 × 10^−12^), regulation of RNA metabolic process (GO:0051252, FDR-1.68 × 10^−11^), regulation of macromolecule biosynthetic process (GO:0010556, FDR- 1.68 × 10^−11^), anatomical structure morphogenesis (GO:0009653, FDR- 8.08 × 10^−11^), and regulation of cellular macromolecule biosynthetic process (GO:2000112, FDR- 8.08 × 10^−11^). The top significantly enriched terms in the cellular component category were: nuclear lumen (GO: 0031981, FDR- 0), nucleoplasm (GO:0005654, FDR- 0.00), plasma membrane (GO:0044459, FDR- 8.69 × 10^−7^), cytosol (GO:0005829, FDR 2.78 × 10^−6^), and so on (Figure 4, Table 2).

The identified target genes were then used to perform pathway prediction using KOBAS (Table 3). The top predicted pathways were PI3K-Akt signaling pathway (*p*-value- 9.99 × 10^−13^), immune system (*p* value- 1.41 × 10^−12^), pathways in cancer (*p* value- 3.05 × 10^−11^), MAPK signaling pathway (*p*-value- 1.02 × 10^−10^), focal adhesion (o value- 1.87 × 10^−10^), Ras signaling pathway (*p* value- 8.38 × 10^−8^), FoxO signaling pathway (*p* value- 8.38 × 10^−8^), and post-translational protein modification (*p* value- 1.25 × 10^−7^). Other important identified pathways included TGF-beta signaling pathway, adaptive immune system, longevity regulating pathway, protein digestion, and absorption and cytokine signaling in the immune system. The list of all pathways related to physiological and metabolic processes enriched by the predicted miRNA responsive target genes is in Supplementary Table S2.

## 3. Discussion

Being transcriptional regulators, miRNAs have been implicated in immune cell differentiation and modulation of immune responses to pathogenic infections [19,20] and also PAMPs such as LPS [21]. However, there is limited information available with regard to the regulatory role of miRNAs in sheep. Here, we have revealed the dynamic nature of serum miRNAs during LPS-induced stress, which may help to better explain regulation of the stress response. To the best of our knowledge, this is the first report exploring the circulatory miRNAs in LPS challenged sheep. A total of 22 miRNAs were significantly DE 4 h post-LPS-challenge compared to the basal pre-challenge levels, implicating their participation in regulating the ovine innate immune response during LPS challenge, and they may serve as stress biomarkers.

The innate immune system of animals provides a first line of defense against pathogenic infections. In parallel, an important relationship between miRNA and innate immunity exists [22]. Exogenous LPS is recognized by cell surface receptors, *TLR4* being the most widely known LPS receptor, which activates downstream intracellular signaling to trigger the innate immune response. The identified DE miRNAs have been reported to modulate the efficiency of the *TLR4* signaling pathway by regulating its several adaptor molecules, proteins, and kinases. The DE miRNAs (miR-1246, miR-200, miR-223, miR-29b, and miR-145) reported earlier in sepsis patients, for example, contribute to regulation of signaling molecules (*IKKα, MAL, TRAF6, MyD88*) in the TLR-4 pathway during LPS exposure [23,24]. Moreover, these miRNAs were reported to regulate innate immunity and cellular functioning of bovine CD14+ monocytes stimulated with LPS [25], and were also upregulated in bovine mammary tissues challenged with *Streptococcus uberis* [26]. Similar induction of miRNAs (miR-223, miR-145. and miR-1246) post-LPS-challenge has also been observed in the equine endometrium [27]. The miR-223, one of the most widely studied miRNAs, positively regulates the proliferation and differentiation of neutrophils, and directly regulates IL-6, chemokines (CXCL2, CCL3) and inflammatory cell recruitment [28], and has already been recognized as a specific and sensitive diagnostic biomarker of sepsis in humans [29,30]. Another induced miRNA (miR-1246) was reported to increase IL-1β and TNF-α production post-LPS-challenge by directly targeting the *ACE2* (angiotensin-converting enzyme-2) gene in pulmonary microvascular endothelial cells [31]. Recently, a study using mesenchymal stem cells reported that miR-1246 regulates *PKA* and *PP2A* by directly targeting their subunits *PRKAR1A* and *PPP2CB*, which leads to *TNFα*-independent *NF-κB* subunit p65-mediated activities and induced the transcription of the pro-inflammatory cytokine IL-6 and chemokines CCL2 and CCL5 [32]. Since cytokines/chemokine level could shape the miRNA signature during infection, it is plausible that the circulatory miRNAs play roles in promoting either tolerance or immunity. The expression of miR-29 was significantly increased (8 fold) at 4 h post-LPS challenge in the present study, which was earlier reported to be induced in LPS-stimulated bubaline blood mononuclear cells [33]. Previously, higher expression of miR-29 was reported in human macrophages infected with *Mycobacterium avium*; miR-29 targets caspase 7 to control cellular apoptosis [34]. Hence, alteration in expression of these miRNAs depicts the induced host response to LPS and highlights their importance in regulating the acute phase response.

Other upregulated miRNAs at 4 h post-LPS-challenge that were identified using PCR array (oar-miR-485-3p, oar-miR-543-3p, and oar-miR-655-3p) were previously reported to activate and regulate the cell survival under stressful conditions by controlling central nervous system [35,36]. The overexpression of miR-485-3p has been implicated in T-cell (CD8+) activation in keratinocytes from cutaneous lupus patients, with a significant increase of *NF-κB/PI3Kδ/PKCø* upregulation, thereby promoting the production of pro-inflammatory cytokines through *AKT* activated *NF-κB* and effector T-cell differentiation [37]. Moreover, miR-485-3p was reported to enhance the *TGF-ß* signaling pathway, which was also one of the top identified pathways in the present study. The miR-655 has been previously documented to induce cell proliferation, and cell migration mediated via *PI3K/Akt* and *ERK* signaling pathways [38]; we observed high miR-655 expression (8.9 fold) post-LPS challenge and in the present study this was also the top enriched pathway. The *PI3K-Akt* pathway was previously enriched by altered miRNA expression in LPS challenged human macrophages [39]. Lastly, the other induced miRNA, miR-543, was previously recognized as an important mediator of multiple types of metabolic stress in humans and is known to upregulate TGF-beta signaling pathway [36], which again was one of the top enriched pathways in the present study.

Besides the above-mentioned miRNAs, two miRNAs (miR-411a-3p/5p, miR-487b-3p) that are known to have anti-inflammatory properties were also induced. Recently, the miR-411a-3p/5p was shown to inhibit inflammation and enhanced recovery in rats following LPS challenge [40]; the scholars suggested that miRNA-411 restrained *NFκB*, and thus ameliorates inflammation and promotes recovery by inhibiting the *JNK* pathway via negatively targeting the pro-inflammatory cytokine IL-18. Similarly, Xiang et al. [41] demonstrated that miR-487b plays a negative regulatory role in the *TLR4* pathway by suppressing LPS-induced expression of specific pro-inflammatory molecules, and thus contributes to the anti-inflammatory response post-LPS challenge.

In contrast, eight miRNAs (miR-31, miR-130b, oar-miR-665-5p, oar-miR-380-5p, oar-miR-323b and c, oar-miR-1197-5p, and oar-miR-154-3p) were significantly reduced 4 h post-LPS-challenge in the resent study. Previously, the inhibition of miR-31 was demonstrated to increase neutrophil adherence to TNF-stimulated human endothelial cells [42]. The miR-31 has mostly been studied using keratinocytes, where it was shown to be regulated by *TGF-b* and collectively induced inflammation by regulating *STK40*, which inhibits TNF-induced NFkB activation [43]. Additionally, downregulation of miR-31-5p has been associated with increased mRNA and protein expression of its target gene, *Foxp3*, which is a crucial transcriptional regulator for the development of regulatory T-cells (Tregs) [44]. Therefore, miR-31 appears to play an essential role in maintaining homeostasis by directly modulating the expansion and function of Tregs, a key event in the regulation of the immune response, in particular regarding immune tolerance. The miR-323-3p was reported to inhibit the *TGF-ß* signaling pathway via targeting SMAD receptors [45], and its inhibition in T cells under bacterial challenge resulted in enhanced clonal expansion of CD8+ T cells, IL-2 secretion, and promoted cell survival [46]. The miR-380-5p is known to suppress *p53*, a key player in the cellular response to stress [47], and thereby regulates cell proliferation. Consequently, downregulation of miR-380-5p will lead to induced expression of *p53* to induce a cellular response and cell proliferation.

Despite accumulating reports of miRNAs in body fluids, little is known about the origins and destinations of these circulating miRNAs. The complex functionality of serum miRNAs is reasonable because they can be carried by various vehicles, including lipoprotein complexes, such as apoptotic bodies, microvesicles, and exosomes. Extracellular miRNAs could be mediators of cell-to-cell signaling and changes in their expression level could reflect damage to source tissues/organs, or aspects of patho-physiological conditions. The differential expression of miRNAs mentioned previously suggests participation in acute phase response via influencing TLR pathways and T-cell activation, which reflects the immune stressed state post-LPS-challenge. Target gene and pathway predictions of the DE miRNAs indicated significant enrichment of gene ontology functional categories, such as development/cellular processes, cell growth and death, immune system (chemokine signaling), signal transduction (*PI3K–Akt* pathway, *MAPK* pathway, and *Ras* signaling pathway), and the nervous system in lambs post-LPS challenge, which demonstrates activation and regulation of defense mechanisms against LPS. A complementary mechanism of both pro- and anti-inflammatory immune responses was observed in present investigation which is likely regulated in part by the identified DE miRNAs. A similar balance of pro- and anti-inflammatory activities was reported in these same lambs at the protein level in our previous study [6].

Furthermore, to establish a relationship between the DE miRNAs, their genomic locations were identified within the ovine genome and their conservation across species was determined using sequence alignment. Based on a search in the miRBase database, and in BLAST with other species, the DE miRNAs were found to be conserved between *Homo sapiens* (has-miR, chromosome 21) and *Bos taurus* (bta-miR, chromosome 14). Intriguingly, most of the identified DE miRNAs were located on chromosome 18 in sheep, which corroborates findings of others who have reported the highest density of miRNA on ovine chromosome 18 (2.40 miRNA loci per Mbp with a total 174 miRNA loci) based on miRNAome analysis of ovine heart, intestine, and muscle tissues [48,49,50]. Collectively, these results highlight the importance of ovine chromosome 18 in terms of unravelling the regulation of ovine innate immune response. Subsequent miRNA clustering analysis identified major miRNA clusters having inter-miRNA distance of <3000 bp on ovine chromosome 18. The clustered miRNAs can be transcribed as a single polycistronic primary transcript and could yield distinct mature miRNAs that can collectively affect the functioning of downstream pathways due to possible coordinated function [51]. The miRNA clusters have high significance in the regulation of physiological processes. Dysregulation of miRNA clusters was shown to be involved in many tumors, such as those of lung, breast, and prostate cancers [18]. Aberrant expression of miRNA clusters can alter cross-talk between inter- and intra-miRNA cluster interactions and have been associated with various pathophysiological events [18]. The identified miRNAs clusters (miR-411/329 and miR-376/655) were previously associated with LPS induced neuronal pathogenesis [52,53]. For example, these clusters initiated microglia–neuron communication in the hypothalamus post-LPS-challenge, which contributes to the neuronal pathogenesis and was also associated with metabolic endotoxemia by activating the ubiquitin–proteasome pathway through TLR4 signaling [54,55]. Thus, the higher expression of these clustered miRNAs implicates their participation in triggering the hypothalamic–pituitary–adrenal axis via influencing TLR-4 signaling; it would be interesting to further explore these clusters for their role in the stress response.

## 4. Materials and Methods

### 4.1. LPS Stress Challenge and Sample Collection

A total of 18 healthy outbred Rideau–Dorset female lambs that were 80–90 days of age were selected for the LPS challenge. These animals were maintained in a pathogen-free environment at the University of Guelph Ponsonby sheep research station (Ponsonby, ON, Canada). The lambs were intravenously (iv) challenged with 1 mL of a 400 ng/kg bolus dose of LPS (*E. coli* O111: B4, Sigma-Aldrich, Darmstadt, Germany) dissolved in 1 mL of saline [6]. Blood was collected from the jugular vein pre-challenge (T0) and 4 h post-challenge (T4) in a 10 mL BD vacutainers serum tube (Becton Dickinson, Franklin Lakes, NJ, USA) to isolate serum miRNAs. All experimental procedures were approved by the University of Guelph Animal Care Committee (AUP # 3436, Jan 14, 2016). Blood was allowed to clot at room temperature (RT) for 40–45 min and serum was isolated by centrifugation at 2000 g for 10 min at RT using a swinging-bucket centrifuge. Isolated serum samples were stored at −80 °C for miRNA isolation.

### 4.2. miRNA Isolation and cDNA Synthesis

A total of 36 serum samples (18 animals at T0 and T4) were thawed on ice and centrifuged at 10,000× *g* for 10 min at 4 °C to remove any debris. The miRNA isolation was performed using the miRNeasy^®^ Serum/Plasma Advanced Kit Qiagen (Hilden, Germany) according to the manufacturer’s instructions. A spike-in control comprised of a known concentration of CE-miR-39-1 (Qiagen) was added as reference miRNA. Briefly, 400 μL of serum was mixed with various volumes of RPL lysis buffer, RPP protein precipitation buffer, and passed through silicone columns. The columns were then washed with RPE and RWT buffers, and miRNA was eluted in 20 µL of nuclease-free water and stored frozen at −80 °C. Further, cDNA was prepared using 5 μL of isolated miRNA from each sample using the miScript II RT Kit (Qiagen, Hilden, Germany) containing miScript HiSpec buffer, 10X nucleic mix, and reverse transcriptase mix. The total reaction mixture of 20 μL was incubated for 1 h at 37 °C and then at 95 °C for 5 min. Finally, cDNA was diluted to 1:11 with nuclease-free water following the given protocol.

### 4.3. miRNA Expression Analysis

Initially, a few candidate miRNAs that were previously associated with LPS-related disorders were analyzed individually in all samples (Table 4). These data were normalized using the Ce-miR-39 spike-in control, and expression levels were determined using the 2−ΔΔCT method [56]. Multiple *t*-test analysis was done and statistical significance was determined using Holm–Sidak correction method using GraphPad Prism v.8.

For large scale profiling of miRNAs, the miScript PCR ovine-specific 384-well (4 × 96) array (Cat # MIVA-001Z, Qiagen, Germany) was employed using a total of 16 samples, which included 12 post-challenge T4 samples and 4 pre-challenge T0 samples. This array consisted of 84 ovine miRNA targets along with controls for data normalization, including 6 miScript PCR controls (1-6 snoRNA/snRNA), a spike-in control for monitoring miRNA isolation (*Caenorhabditis elegans* miR-39; Ce), a reverse transcription control assay (miRTC), and positive PCR-controls (PPC) to monitor PCR inhibitors. The reaction mixture of 10 µL containing premix of cDNA, miScript Universal Primer, SYBR Green PCR Master Mix (BioRad, California, USA), and RNase-free water was added in each of 384-wells in the array. The reaction was amplified and quantified using the SYBR-Green chemistry on a viia7 qPCR instrument (ABI, USA). The expression data were exported into a Microsoft Excel spreadsheet and data were analyzed using the Qiagen Gene Globe data analysis center (https://geneglobe.qiagen.com/us/), and fold-change was calculated using the ∆∆CT method of relative quantification [56]. Only miRNAs with CT < 35 were included in the analysis, and miRNA expression levels were normalized using the global mean normalization method [57]. This method automatically calculated a global CT means for the miRNA targets that were commonly expressed in all the samples being analyzed after an initial calibration with the exogenous cel-miR-39 spike-in control. The final miRNA expression values were generated in a biologically meaningful way as fold-regulation (FR) which represents fold-change values. For FR, fold-change values less than 1, meaning that the miRNA was down-regulated, were transformed by calculating the negative inverses. Statistical analysis involving t-test comparisons between the control (T0) and treated (T4) time points was performed using the Qiagen Gene globe analysis software.

**Table 4:** Details of forward primer sequences used for individual miRNA analysis by qPCR.

miRNA	Forward Primer (5′-3′)	Annealing Temperature	References
miR-145a	GTCCAGTTTTCCCAGGAATCCCT	60 °C	[58]
miR-130b	AGCAGGCAGTGCAATGATGA	60 °C	
miR-145	GTCCAGTTTTCCCAGGAATCC CT	60 °C	
miR-223	CCTGTCAGTTTGTCAAATACC CCA	60 °C	
miR-1246	GAATGGATTTTTGGAGCAGGA A	60 °C	
miR-31	GGAAGGCAAGATGCTGGCA	60 °C	
miR-29b	GCGTAGCACCATTTGAAATC	60 °C	

### 4.4. Target Gene Prediction and Pathway Analysis

The target genes of the differentially expressed (DE) miRNAs with significant (*p* < 0.05) FR were predicted through three database tools: TargetScan (http://www.targetscan.org), mirDB (http://www.mirdb.org/index.html), and Miranda target prediction tools (http://www.microrna.org/microrna/getGeneForm.do). A stringent selection criteria of target genes for each tool was applied: cumulative weighted context++ score <−0.4 for Targetscan; target score >70 for miRDb; and mirsvr score <−0.1 for Miranda. The commonly identified target genes, at least between two tools, were considered for functional analysis. To facilitate the interpretation of gene targets and aid in the understanding of the potential function of the miRNAs, enrichment analysis for gene ontology (GO) annotation (molecular function, cellular component, and biological process) was performed using WebGestalt (http://www.webgestalt.org/#, version 2019), and pathway enrichment analysis was performed using KOBAS 3.0 (http://kobas.cbi.pku.edu.cn/kobas3) with Benjamin and Hochberg false discovery rate (FDR) < 0.05 correction. 

### 4.5. Genomic Localization and Cluster Analysis

The DE miRNAs were queried in the recent version of miRbase (release 12 March 2018, miRbase.org) to retrieve sequences and chromosomal site location in sequence map of *Ovis aries* (Oar_v4.0). Further, the clusters of DE miRNAs, where miRNAs are located within <3000 bp, were identified using the MetaMirClust database (http://fgfr.ibms.sinica.edu.tw/MetaMirClust/) and miRbase; the MetaMiRClust database provides comprehensive information about the conservation of miRNA clusters in various animal genomes [59].

## 5. Conclusions

Although much remains to be learned about the acute-phase response, emerging evidence highlights the importance of miRNAs in maintaining homeostasis during pathogenesis. The present findings elucidated the interactions among DE miRNAs and their target genes, and highlighted the interesting role of chromosome 18 and its miRNA-clusters (miR-379/495 and miR-411/329) in resolution of the inflammatory response. The DE serum miRNAs identified following LPS exposure may serve as promising stress biomarkers and were well aligned with other analyzed cytokine and chemokine biomarkers. Furthermore, the identified DE miRNAs presented as a balance of pro- and anti-inflammatory drivers, which is likely required for efficient elimination of infectious agents and to control collateral immune-mediated tissue damage. Further studies are warranted to explore roles of chromosome 18 and miRNA clusters within to better understand their contributions to the ovine stress response.

## Figures and Tables

**Table 1:** Differentially expressed ovine serum miRNA 4 h post-systemic LPS challenge. * indicates significance at *p* < 0.05.

Mature ID	Fold-Regulation	*p*-Value	Mature ID	Fold-Regulation	*p*-Value
Upregulated				Downregulated	
oar-miR-369-3p	140.89	0.011 *	oar-miR-154b-3p	−2.41	<0.001 *
oar-miR-495-3p	57.94	0.055 *	oar-miR-323b	−2.6	0.002 *
oar-miR-376a-3p	32.09	0.050 *	oar-miR-323c	−2.47	0.004 *
oar-miR-543-3p	22.15	0.002 *	oar-miR-380-5p	−2.78	0.002 *
oar-miR-668-3p	14.85	0.022 *	oar-miR-665-5p	−2.54	0.001 *
oar-miR-329a-3p	13.22	0.028 *	oar-miR-1197-5p	−2.61	0.002 *
oar-miR-487b-3p	12.29	0.060			
oar-miR-485-3p	10.29	0.077			
oar-miR-655-3p	8.99	0.016 *			
oar-miR-411a-5p	6.72	0.050 *			
oar-miR-154a-3p	3.99	0.027 *			

**Table 2:** List of the top 10 identified gene ontology (GO) terms and false discovery rate (FDR) using target genes of upregulated miRNAs at 4 h post-systemic ovine LPS challenge.

Gene Set	Description	FDR
**Biological Processes**		
GO:0010468	Regulation of gene expression	1.48 × 10^−12^
GO:0051252	Regulation of RNA metabolic process	1.68 × 10^−11^
GO:0010556	Regulation of macromolecule biosynthetic process	1.68 × 10^−11^
GO:0019219	Regulation of nucleobase-containing compound metabolic process	1.93 × 10^−11^
GO:0009653	Anatomical structure morphogenesis	8.08 × 10^−11^
GO:2000112	Regulation of cellular macromolecule biosynthetic process	8.08 × 10^−11^
GO:0009889	Regulation of biosynthetic process	9.23 × 10^−11^
GO:0031326	Regulation of cellular biosynthetic process	1.06 × 10^−10^
GO:0072359	Circulatory system development	1.16 × 10^−10^
GO:0051254	Positive regulation of RNA metabolic process	1.31 × 10^−10^
**Cellular Component**		
GO:0031981	Nuclear lumen	0.00
GO:0005654	Nucleoplasm	0.00
GO:0097458	Neuron part	2.47 × 10^−7^
GO:0044459	Plasma membrane part	8.69 × 10^−7^
GO:0005829	Cytosol	2.78 × 10^−6^
GO:0120025	Plasma membrane bounded cell projection	4.86 × 10^−6^
GO:0042995	Cell projection	4.86 × 10^−6^
GO:0044456	Synapse part	6.34 × 10^−6^
GO:0098978	Glutamatergic synapse	1.23 × 10^−5^
GO:0036477	Somatodendritic compartment	1.38 × 10^-5^
**Molecular Functions**		
GO:0140110	Transcription regulator activity	1.57 × 10^−8^
GO:0003700	DNA-binding transcription factor activity	1.64 × 10^−8^
GO:0000981	DNA-binding transcription factor activity, RNA polymerase II-specific	1.64 × 10^−8^
GO:0000977	RNA polymerase II regulatory region sequence-specific DNA binding	2.41 × 10^−8^
GO:0043565	Sequence-specific DNA binding	2.41 × 10^−8^
GO:0001012	RNA polymerase II regulatory region DNA binding	2.41 × 10^−8^
GO:0000976	Transcription regulatory region sequence-specific DNA binding	3.16 × 10^−8^
GO:0001067	Regulatory region nucleic acid binding	3.16 × 10^−8^
GO:0044212	Transcription regulatory region DNA binding	1.57 × 10^−8^
GO:0140110	Transcription regulator activity	1.64 × 10^−8^

**Table 3:** List of the top 10 enriched pathways with corrected *p*-values and corresponding databases, identified using target genes of upregulated miRNAs at 4 h following systemic ovine LPS challenge.

S.No	#Term	Corrected *p*-Value (FDR)	Database
1	PI3K-Akt signaling pathway	9.99 × 10^−^^13^	KEGG Pathway
2	Immune System	1.41 × 10^−^^12^	Reactome
3	Pathways in cancer	3.50 × 10^−^^11^	KEGG Pathway
4	MAPK signaling pathway	1.02 × 10^−^^10^	KEGG Pathway
5	Focal adhesion	1.87 × 10^−^^10^	KEGG Pathway
6	Ras signaling pathway	8.38 × 10^−^^10^	KEGG Pathway
7	FoxO signaling pathway	8.83 × 10^−^^8^	KEGG Pathway
8	Rap1 signaling pathway	1.13 × 10^−^^7^	KEGG Pathway
9	Post-translational protein modification	1.25 × 10^−^^7^	Reactome
10	Signal Transduction	1.92 × 10^−^^7^	Reactome

## Supplementary Materials

Supplementary materials can be found at https://www.mdpi.com/1422-0067/21/21/7920/s1.

## Abbreviations

LPS	Lipopolysaccharide
IV	Intravenously
miR	MicroRNA
oar-mir	Ovine MicroRNA
DE	Differentially Expressed
FR	Fold regulation
MAPK	Mitogen-Activated Protein Kinase
PI3K	Phosphoinositide 3-kinases
FDR	False discovery rate
